# Present Systems and the Impending Education

**Published:** 1883-10

**Authors:** A. H. Thompson

**Affiliations:** Topeka, Kansas.


ARTICLE III.

### PRESENT SYSTEMS AND THE IMPENDING EDUCATION

BY A. H. THOMPSON, D. D. S., TOPEKA, KANSAS.

(Read before the Missouri State Dental Association, at Sweet Springs Mo., July 11, 1883.)

As a preliminary step to the consideration of the matter in hand, let us review the varieties of systems, with their varying quantities and qualities of education, which are found in our midst to-day, and which pass under the name of education. We find the genius of education sailing under different banners and presenting itself in a variety of guises, which we will endeavor to briefly notice and classify.

The *first*, then, beginning with the lowest organized species in the *genus* of educational institutions, so called, is the mere diploma mill,— the sheepskin shop,—which is, in fact, in no sense educational at all, but exists as a mere libel upon the fair name of education. All respectable practitioners of the profession, unite in condemning and execrating this excresence, and in denouncing and ostracising its perpetrators. There is but one opinion concerning this class of institutions, and they are mentioned but to be condemned. The wheels of the law have been fully set in motion against them, and all men cry—let them speedily be suppressed!

The *second* grade, which may be called a step higher on the moral educational ladder, is the college which grants degrees for "merit alone," without regard to time of attendance upon lectures or instruction of any sort, or, indeed, without any such attendance at all, provided the examinations, so called, are passed. This system is attractive, theoretically, and commends itself to a large body of superficial thinkers and would-be reformers, but practically it is worthless. Theoretically and in the abstract, the system seems fair, in that it would reward that large body of well-informed, capable practitioners, prominent in the profession, who are studious, energetic, and worthy men, and who better deserve the distinction of receiving the degree than many who receive it by regular attendance upon lectures and graduation by the colleges. But, on the other hand, when the system is put into practice, it is not the worthy and deserving men who obtain the degree; they do not, as a rule, come forward and apply for it, and if they did, their very modesty and honesty would lead to their discomfiture and undeserved failure before the examiners—*if* the theoretical strictness of examination was maintained. But it is the unworthy pretender who applies, and whose lack of knowledge is more than compensated for by his assurance, and he comes out with flying colors—a disgrace alike to the system and to the profession. In addition to the difference between modesty and cheek, as counting for or against success, is the laxity necessary to the success of the system, and the absence of the conscientious application even of its merits. It is this inherent necessity for laxity which condemns the system and renders it impracticable and dishonest. It is easy to reply that, "*If* the 'merits alone' system were conscientiously applied," etc. It is the *if* that condemns the system. It is not—cannot be practically sustained in its theoretical purity. It is not too much to say that is it beyond human power to make it strict, or, indeed, to make any system strict and conscientious which depends solely and alone upon examinations as a test of the ability of the student. Injustice alike to the worthy and the unworthy is sure to result and the honors to be misplaced.

The *third* system is a full step higher in the scale, although bearing a strong family resemblance to the last described. It is that custom which obtains with many colleges even yet, although it is now, fortunately, becoming obsolete and disreputable, of considering five years practice, or experience in the profession as an equivalent for the attendance upon a course in the college, and the passing of the terminal examinations. This is just as much better than the preceding system, as that one course of lectures is better than none at all; and the degree is also truthful in that it certifies to the enjoyment of *some* opportunity, at least, for the acquisition of knowledge. But the one course represents all the scientific training the graduate possesses, and he cannot possibly have been fitted in that brief period for intelligent practice. The stock of intellectual furniture acquired in that short time must necessarily be limited. Of course, this system has the advantage that many of the exceptionally worthy, theoretical cases do attend the one course of lectures and graduate with honor to themselves and their *alma mater*. Thus far, the system is right and honest, and the practice sustains the theory. But the ideal cases are, unfortunately, in the minority; and the unworthy, those who are unfit to wear the honors, constitute the majority of the five-years-and-one-course class of graduates. The custom is unfair to those who earn the degree, and it gives birth to most of the graduated quacks. In response to the demands of the reform movement in the profession, it is being gradually abandoned.

The *fourth* system in order is more than a step higher than the preceding, and marks the emergence into the full daylight of conscientious educational work. It is that of requiring attendance upon at least two courses of lectures before graduation and the bestowal of the degree. It imposes the opportunities of two yearly courses of didactic lectures, clinical and practical teaching, and other scientific instruction in medical or dental colleges where such opportunities are afforded. The degree then assumes that the possessor has been subjected to the best opportunity, and that he presumably possesses the attainments and abilities required. This system is, of course, more fruitful than the single course of creditable and useful results. The difference is readily noticed between the material the two systems produce. The two-year students, raw and uncouth as many of them are, are better fitted for a successful and honorable career in the practice of the profession than the average practitioner of five or more years' experience, who obtains the parchment after one winter spent in college. The reason is obvious. The two-years' men are generally young men who attend college mainly for study and instruction, and that during the plastic period of life, before the mind is cramped by confined habits or the brain becomes stiffened and unimpressionable by age. They there, perforce, acquire habits of study and imbibe of the spirit of investigation which surrounds them, and, if ambitious, will cultivate those habits when they enter upon practice and become honorable and useful members of the profession. But the practitioner, as a rule, goes merely to obtain the degree. He has studied little in his time, has never observed that he needed to study, and protests against the necessity of beginning now. But he must acquire some practical knowledge in order to be enabled to "pass," so he crams for that purpose and that alone. The ambitious two-years student is the proof of the wisdom of the system. His attainments are broad and varied, and he is likely to carry the impres sions of the inspiring associations of college life far into after years, the memory of those days lingering as a perpetual impulse to do better and achieve more.

The *fifth* system, the next higher in merit, and a full step beyond the preceding, is that of dental lectures in connection with medical colleges. We place this system above that of the purely dental colleges, because of the growing sentiment manifested in its favor, and the growing conviction throughout the intelligent and thoughtful classes of the profession that such combined institutions are on the whole, better able to confer the required scientific education than the purely dental colleges. There has been much discussion and controversy of late years as to the comparative merits of the medico-dental and the purely dental systems, but there is little doubt of the growing favor of the joint system. There is a spreading belief, penetrating even to the mediocre ranks of the profession, that medical colleges are the better qualified to furnish that broader medical knowledge of which the average practitioner stands so much in need; and the advanced minds are beginning to admit that the superior specialists found in the medical schools are naturally better able to furnish complete instruction in the broad, underlying fundamental principles upon which we can alon efound a perfect education. The dental colleges provide a special education which, with its tendency toward becoming too special, is too prone to ignore that breadth of knowledge necessary to full instruction in the principles. Take anatomy and physiology, for instance; every one knows that the dental student should be as broadly grounded in these principles as the medical student; and yet the dental colleges concentrate their instructions in them to the immediate necessities of the dental surroundings, and the dental student acquires no knowledge of their breadth and and general importance. It is this fact that is at the root of the sentiment which is permeating the profession to-day.

The *sixth* and last system which remains to notice, is the highest which our education has yet attained, and is head and shoulders above everything else within the experience of the profession. We refer to those dental colleges connected with the medical departments of great universities, which require the student to pass a preliminary examination before matriculation and admission. This is universally conceded to be the most prudent, wise, and dignified course of procedure that any system has yet inaugurated, and that it will be the most fruitful of good results. It approaches that ideal system where the perfect standard can be maintained without fear or favor. The reformers and their followers have been urging all the colleges to adopt the method, but there does not seem to be any others which can rise above the necessity of turning out graduates. It is greatly to be regretted that the idea is not contagious; but we are in no immediate danger of a revolution from this cause, much as we could wish it. The idea is too radical, too violent, too perilous for the conservative type of colleges. But whether tasteful to the college proprietors of this country or not, whether they ever adopt the innovation or not, the education of the future will necessarily include such a factor in its structure. Preliminary examinations will be a fundamental clause in the constitution of that system which will soon dawn upon us, and toward which we are looking so hopefully. It will be a necessary means toward the selection of the material from which to construct the dental and oral profession of the future. A just discrimination between the ignoramus and the educated applicant for admission to our ranks will be the first step toward the purification of the stream which replenishes our ranks and which will make our future.

Having thus reviewed the systems of dental education which we have with us to-day, and considering well their power for evil or for good, the question arises, what of the future? Like the mound-builder of old mounted on his lookout or the bluff whence he could see far and near, standing as we do in the last but one decade of the nineteenth century, with the past behind us with its lessons of experience, the present around us with its hopes and fears, we must ask the anxious question—What of the future?

We notice, first, that, the education of the future must be developed from, must be the offspring of, the education of the present. A revolution—a radical, sweeping change —is impossible. Something will be developed from one or more of the present systems. The existing confusion cannot last, and it is undesirable that it should. There is "confusion worse confounded" in the present medley, and a discontent with all its growing and shaping until it will bring something definite from the chaos, or end in an utter nihilism which will destroy all education. Or, perhaps, the conflict might go on until the worst system possible will come out ahead, or the worst results possible will follow. But of this we have no fear. There is leaven at work which will purify the whole lump and bring order out of chaos. There are some signs of the times which indicate which way the wind blows, and which tell us that a force is forming which will bring harmony at last, and we hope, before it is too late. There will be *unity*, at least; but what form that unity will take, and what will be the impending education toward which we are looking so anxiously, we cannot yet predicate. But the force at work will yield us something definite, which will most probably be a satisfactory solution of the much-discussed problem of education.

The force to which we refer, which will exercise so potent an influence upon the education of the future, is the State Boards of Examiners in States which have and will have laws controlling the practice of dental surgery. This is a a factor in the problem of dental education, the introduction of an element into its solution, the importance of which has scarcely begun to be suspected, and whose power to mold the education of the future no one can estimate. It will not be long before every organized State will adopt a law controlling the entrance of young men in the practice of the profession. Then will come the consummation of the tendency to union and harmony manifested by the recent conference of State Boards of Examiners, and a uniform law and a uniform standard will ultimately prevail. In view of this, the organization of the conference of State Boards assumes the aspect as being one of the most momentous events, if not the most momentous and important occurrence, which has happened in the history of dental education in this country within the memery of the present generation. It is an event so pregnant with meaning as to be second in importance only to the organization of the first dental college in this country. That conference or union of State Boards, if it is made permanent, as it undoubtedly will be, is the power which will dictate the education and control the standard of the future. On it the impending education depends for shape and features. It will mold the standard to meet the requirements of the profession, and the power, coming as it will directly from the ranks, will voice the sentiments of the profession. It will raise or lower the standard for admission to colleges and for graduation; it will alter the curriculum and dictate the methods of education in accordance with the popular will, and do all with a ruthless hand, because it holds the keys of admission to the practice of the calling. It will become the practical custodian of all things pertaining to education, and will rule with a pitiless sway, deaf alike to the appeals of starveling colleges and the ravings of fanatical reformers. Why, even now, we read of Boards going behind the diplomas of some colleges and examining the possessors of them! The conference will then rule securely and firmly, and it becomes at once apparent that this will be the best possible solution of the vexed question of education. It will cause no suffering except to the unworthy, and do injustice to none. The colleges fit to live can live, but the diploma mills and other workers of iniquity will go down before this irrisistible and merciless engine of reformation. The better colleges can doubtless easily conform to the requirements of the conference, for the popular voice of the profession to-day is not unreasonable, and that will be the power which will make itself felt. But if they will not conform they must go down, for from the decisions of this power there will be no appeal. It will be the court of last resort. It will stand upon the merits of the applicants and the good of the profession, and will judge without fear or favor. But the fruit of it all will be that we shall be elevated, purified, blessed! for the purposes and workings of the conference can only conduce to good. But we are yet unapprised as to the form and features which this new —the impending—education which will be brought about by this new force will assume. We cannot predict, for instance, what will be the standard of admission to practice which will be adopted by the conference; but we can safely assume that the common-sense suggestion will occur to and prevail with these practical men that it shall be low at first, and from thence be elevated gradually. Indeed, it must be low at first, for the standard of the average qualifications of the examiners is itself low. Until the examiners are themselves educated—and this will take time—it is safe to assume that the sliding scale will be employed, which will be at once safe and practical. It is probable, also, that the general aspect of the curriculum required by the conference will be largely dental at the first, with a gradual tendency toward the medico-dental system. In the lapse of time we will then reach our ultimate destination of fusion with the medical education, which is the goal and fate of all educated specialities of human medicine. Indeed, to-day, as our education becomes higher it becomes more medical, we acquire greater knowledge of medical sciences, and we become more affiliated with medical men and the medical profession. In view of this, ultimate fusion seems inevitable and is not undesirable. We will then be not less dentists, but more of physicians. In all the pages of progress of recent years we notice that as dentists become more educated they become more medical. They are obliged to refer to medical text-books for their knowledge of the fundamental sciences of our calling, and in becoming broader scientists they become more of physicians. It seems, therefore, self-evident that as a separate branch of the healing art we cannot hope long to retain our independence of the Maternal Medicine.

But whatever the system of the future may be, either immediately or finally, we may assure ourselves that it will be the golden era, and that the dental profession will then be something of which its then members may be proud. It will be something at once thoroughly scientific and thoroughly honest and practical.—*Missouri Dental Journal*

